# Identification of the initial nucleocapsid recognition element in the HIV-1 RNA packaging signal

**DOI:** 10.1073/pnas.2008519117

**Published:** 2020-07-09

**Authors:** Pengfei Ding, Siarhei Kharytonchyk, Alexis Waller, Ugonna Mbaekwe, Sapna Basappa, Nansen Kuo, Heather M. Frank, Christina Quasney, Aaron Kidane, Canessa Swanson, Verna Van, Mitali Sarkar, Emily Cannistraci, Ridhi Chaudhary, Hana Flores, Alice Telesnitsky, Michael F. Summers

**Affiliations:** ^a^Department of Chemistry and Biochemistry, University of Maryland, Baltimore County, Baltimore, MD 21250;; ^b^Howard Hughes Medical Institute, University of Maryland, Baltimore County, Baltimore, MD 21250;; ^c^Department of Microbiology and Immunology, University of Michigan Medical School, Ann Arbor, MI 48109

**Keywords:** HIV-1, genome, RNA, packaging, nucleocapsid

## Abstract

Selective packaging of the HIV-1 genome during virus assembly is mediated by interactions between the dimeric 5ʹ-leader of the unspliced viral RNA and the nucleocapsid (NC) domains of a small number of assembling viral Gag polyproteins. Here, we show that the dimeric 5′-leader contains more than two dozen NC binding sites with affinities ranging from 40 nM to 1.4 μM, and that all high-affinity sites (*K*_d_ ≲ 400 nM) reside within a ∼150-nt region of the leader sufficient to promote RNA packaging (core encapsidation signal, Ψ^CES^). The four initial binding sites with highest affinity reside near two symmetrically equivalent three-way junction structures. Unlike the other high-affinity sites, which bind NC with exothermic energetics, binding to these sites occurs endothermically due to concomitant unwinding of a weakly base-paired [UUUU]:[GGAG] helical element. Mutations that stabilize base pairing within this element eliminate NC binding to this site and severely impair RNA packaging into virus-like particles. NMR studies reveal that a recently discovered small-molecule inhibitor of HIV-1 RNA packaging that appears to function by stabilizing the structure of the leader binds directly to the [UUUU]:[GGAG] helix. Our findings suggest a sequential NC binding mechanism for Gag-genome assembly and identify a potential RNA Achilles’ heel to which HIV therapeutics may be targeted.

## Significance

Understanding the molecular determinants of retroviral genome packaging is important for drug discovery and development of vectors for gene delivery. We show that the HIV-1 leader, which contains the RNA elements necessary for genome packaging, binds approximately two dozen copies of the cognate NC protein with affinities ranging from ∼40 nM to 1.4 µM. Binding to the four highest-affinity “initial” binding sites occurs with endothermic energetics attributed to NC-induced localized RNA melting. Mutations that stabilize these sites inhibit NC binding in vitro and RNA packaging in transfected cells. A small-molecule inhibitor of RNA packaging binds specifically to the initial NC binding sites and stabilizes the RNA structure. Our findings identify a potential RNA Achilles’ heel for HIV therapeutic development.

Selective incorporation of authentic viral genomes into assembling virions is essential for viral replication (1). Like all retroviruses except the spumaviruses, the HIV type 1 (HIV-1) specifically packages its positive-sense, unspliced genomic RNA (gRNA) in the form of a noncovalently linked dimer (2–6). Both strands of the dimeric gRNA are used for strand transfer-mediated recombination during reverse transcription (7–9), which enhances HIV-1 fitness by maintaining genetic integrity and promoting genetic diversity under natural or chemotherapeutic stresses (10, 11). Although the full-length gRNA only accounts for <1% of all cytosolic RNAs (including viral spliced RNAs and nonviral RNAs), the dimeric gRNA exists in >90% of all newly assembled progeny virions (12). This highly selective packaging process is mediated by specific interactions between a *cis*-acting element of the HIV-1 gRNA and the nucleocapsid (NC) domain of the major structural polyprotein Gag (13–15).

The HIV-1 packaging signal is located within the 5′-leader (5′-L) of the viral genome (2, 6, 16), which is among the most conserved regions of the viral RNA (3, 17). The leader contains discrete stretches of nucleotides with independent and sometimes overlapping functions: a *trans*-activation response region (TAR) that stimulates transcription, a primer binding site for initiation of reverse transcription (PBS), a dimer initiation site that promotes genome dimerization (DIS), the major splice donor site (SD), a polyadenylation signal (polyA), and the *gag* start codon (AUG) (3, 18–22). The major determinants for HIV-1 genome packaging were initially assigned to a relatively short segment surrounding conserved residues predicted to form a hairpin (called Ψ) (23–25), and later expanded to include a much larger region of the leader (26–28). Combinations of nucleotide accessibility mapping and mutagenesis studies indicate that the secondary structure of the dimeric 5′-L that is selected for packaging differs from that of the monomer (6, 29–31). Although gRNA versus mRNA functions were originally thought to be modulated by a riboswitch-like mechanism, in which the function of a single RNA transcript is modulated by changes in its structure (29, 32, 33), it now appears transcript dimerization and fate are controlled at the level of transcription by heterogeneous start site usage. Thus, transcripts that begin with two or three sequential 5′-guanosines form monomers in vitro and are retained in cells for splicing and/or translation, whereas those that begin with a single 5′-guanosine form dimers and are selectively packaged into virions (34–36). A combination of mutagenesis, NMR, and RNA packaging experiments identified a region of the leader sufficient for packaging heterologous vectors into assembling virions (called the core encapsidation signal, Ψ^CES^) (37), which has been shown by NMR to adopt a tandem three-way junction structure (30).

gRNAs are recognized by the NC domain of Gag, which contains two copies of a conserved “CCHC”-type zinc finger domain that are critical for genome packaging (25, 38). Structural studies of NC bound to several 5′-L fragments all show that tight binding is mediated by a combination of electrostatic interactions and sequestration of exposed guanosine bases within hydrophobic clefts of the zinc fingers (39–42). Confocal studies indicate that genomes are sequestered at plasma membrane assembly sites by a small number of Gag proteins, probably a dozen or fewer, and that this initial complex nucleates further Gag recruitment and virus assembly (43, 44). In vitro assembly of immature virus-like particles can also be stimulated by the presence of the dimeric [Ψ^CES^]_2_ RNA (45).

The present studies focused on identifying the mechanism that guides assembly of Gag on the HIV-1 packaging signal. We found that the HIV-1 dimeric 5′-L contains more than two dozen NC binding sites with affinities spanning from ∼40 nM to 1.4 µM, and that binding sites with highest affinity map within the [Ψ^CES^]_2_ dimer. Our studies also revealed that initial binding to the highest-affinity binding sites occurs with localized and concomitant unwinding of a short and dynamic helical structure located near a tandem three-way junction structure, and that the lability of this helix is important for genome packaging and viral replication. Implications of our findings for virus assembly and antiviral development are presented.

## Results

### Dimeric 5′-L Binds More than Two Dozen NC Proteins with Affinities that Vary by an Order of Magnitude.

**Fig. 1.**
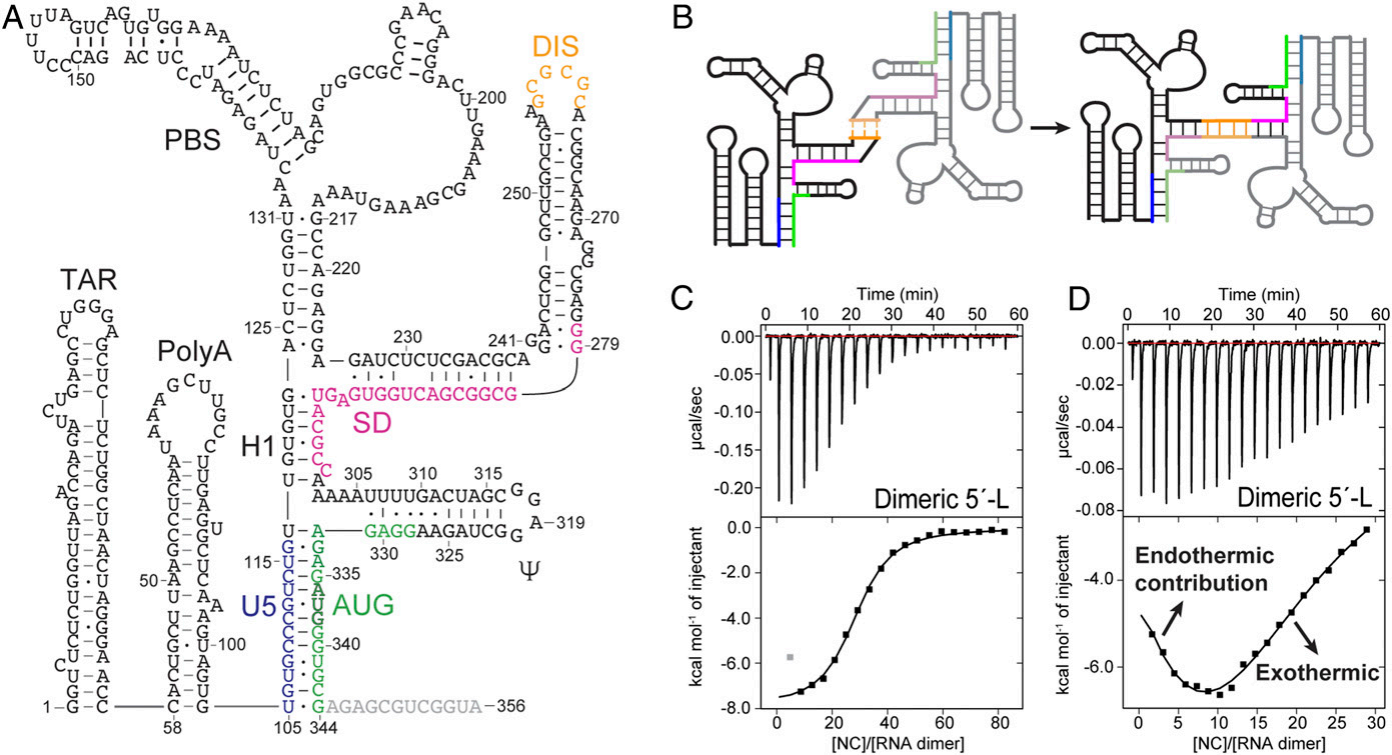
The dimeric 5′-L contains more than two dozen NC binding sites. (*A*) Secondary structure of the HIV-1 5′-L in the dimer promoting conformation. The U5, DIS, SD, and AUG sequences are colored in blue, yellow, magenta, and green, respectively. (*B*) Dimerization of 5′-L initiated by DIS “kissing” interaction and stabilized by extensive intermolecular interactions. ITC isotherms for the dimeric 5′-L titrated by NC with high (*C*) or low (*D*) protein-to-RNA ratios. The gray calorimetric data point was not included in data fitting.

The 5′-L of the HIV-1 RNA genome includes the 5′-untranslated region and the first few codons of *gag* (residues 1 to 356 for HIV-1_NL4-3_) (Fig. 1*A*) (29). Dimerization is initiated by intermolecular base pairing between the palindromic “GCGCGC” sequences within the DIS regions of two 5′-L RNAs, followed by the formation of extensive intermolecular interactions (Fig. 1*B*) (31). In the dimer-promoting conformation of the full-length 5′-L, residues of the unique-5′ (U5) region base pair with residues near the *gag* start site (U5:AUG interactions) leaving residues A345–A356 as a single-stranded unstructured region (Fig. 1*A*). Previous studies have shown that truncation of these disordered 3′-residues downstream of the AUG start site shifts the equilibrium of the 5′-L from the monomer to the dimer conformation (31, 37). Thus, the leader construct we used in the current study contains residues 1 to 344 (5′-L_344_ or 5′-L), which readily forms dimers when incubated under buffer conditions with physiological-like ionic strength (PI buffer, 140 mM KCl, 10 mM NaCl, 1 mM MgCl_2_, pH 7.0) (see Fig. 4*D*). All RNA constructs used in this study are derivatives of this dimeric 5′-L with extended intermolecular base pairing (*SI Appendix*, Fig. S1).

Previous isothermal titration calorimetry (ITC) studies from our laboratory showed that the dimeric 5′-L gave rise to a bipartite NC-binding isotherm under PI buffer conditions (29, 37). Initially, high-affinity exothermic bindings were observed, followed by a subsequent endothermic event associated with weak, nonspecific NC binding and NC-induced RNA unfolding at higher NC-to-RNA ratios. Higher concentrations of Mg^2+^ (e.g., 5 mM) were later found to eliminate the nonspecific binding and unfolding (29, 30). Therefore, all of the present ITC studies were performed with a PI buffer containing 5 mM MgCl_2_, allowing for accurate fitting of the high-affinity specific NC binding data. Interestingly, while defining the high-affinity exothermic and the Mg^2+^-sensitive nonspecific binding modes, we noticed that the first one or two data points were not consistent with a simple exothermic high-affinity binding event. The first two data points were typically excluded in prior ITC studies of NC binding to the HIV-1 leader (29, 37). However, these unusual data points were also observed in the titrations with 5 mM Mg^2+^ (gray data point in Fig. 1*C*), and an early endothermic binding event was clearly detected using reduced NC-to-RNA ratios (Fig. 1*D*). Saturation of this endothermic binding at an NC-to-RNA ratio of ∼10 suggests the presence of approximately four NC binding sites with affinities significantly higher than those of the exothermic binding sites.

Fitting of the ITC data using a single-set-of-sites model with the initial “outlier” data points deleted (gray data point due to endothermic binding in Fig. 1*C*) indicates that the dimeric 5′-L binds 28 ± 1 NC molecules with an average *K*_d_ of 880 ± 30 nM (Table 1). This average NC-binding affinity of the dimeric 5′-L is much lower than that of the well-studied Ψ-hairpin apical loop, which contains a single high-affinity site (*K*_d_ = 320 ± 30 nM) (Table 1), indicating that NC binding to the intact, dimeric leader occurs with heterogeneous binding affinities. Consistent with this hypothesis, an RNA construct comprising the TAR-PolyA tandem hairpin was found to have two weak NC binding sites with an average *K*_d_ of 1,320 ± 140 nM (*SI Appendix*, Fig. S2*B*). A leader construct without the PBS region (replaced by a “GAGA” tetraloop, 5′-L^ΔPBS^) binds 23 ± 1 NC with decreased average *K*_d_ (760 ± 50 nM; *SI Appendix*, Fig. S2*A*), suggesting that the PBS region only has two to three weak NC binding sites. Thus, the more than two dozen specific NC binding sites of the dimeric 5′-L are notably heterogeneous in terms of both binding energetics and binding affinities.

**Table 1.** NC binding stoichiometry and affinity of HIV-1 5′-L RNA constructs

Constructs	Endothermic		Exothermic	
	*N*	*K*_d_, nM	*N*	*K*_d_, nM
5′-L	N/D	N/D	28.0 ± 1.1	880 ± 30
5′-L^ΔPBS^	N/D	N/D	22.6 ± 1.1	760 ± 50
Ψ^CES^	4.0 ± 0.9	45 ± 19	15.9 ± 0.9	330 ± 20
Ψ^T−3WJ^	2.8 ± 0.2	43 ± 18	5.2 ± 0.2	430 ± 50
Ψ^3WJ−1^	2.2 ± 0.2	28 ± 15	2.7 ± 0.3	320 ± 180
DIS	N/A	N/A	6.6 ± 0.2	340 ± 30
Ψ^HP^	2.3 ± 0.2	26 ± 21	2.8 ± 0.3	1,540 ± 240
Ψ^HP-stable^	N/A	N/A	1.2 ± 0.3	300 ± 150
Ψ-fragment	N/A	N/A	1.8 ± 0.1	450 ± 30
Ψ-apical loop	N/A	N/A	1.2 ± 0.1	320 ± 30
TAR-PolyA	N/A	N/A	1.9 ± 0.1	1,320 ± 140

N/A, not applicable; N/D, not determined. Data are represented as mean ± SEM (*n* = 3).

### High-Affinity NC Binding Sites Reside within the Core Encapsidation Signal.

**Fig. 2.**
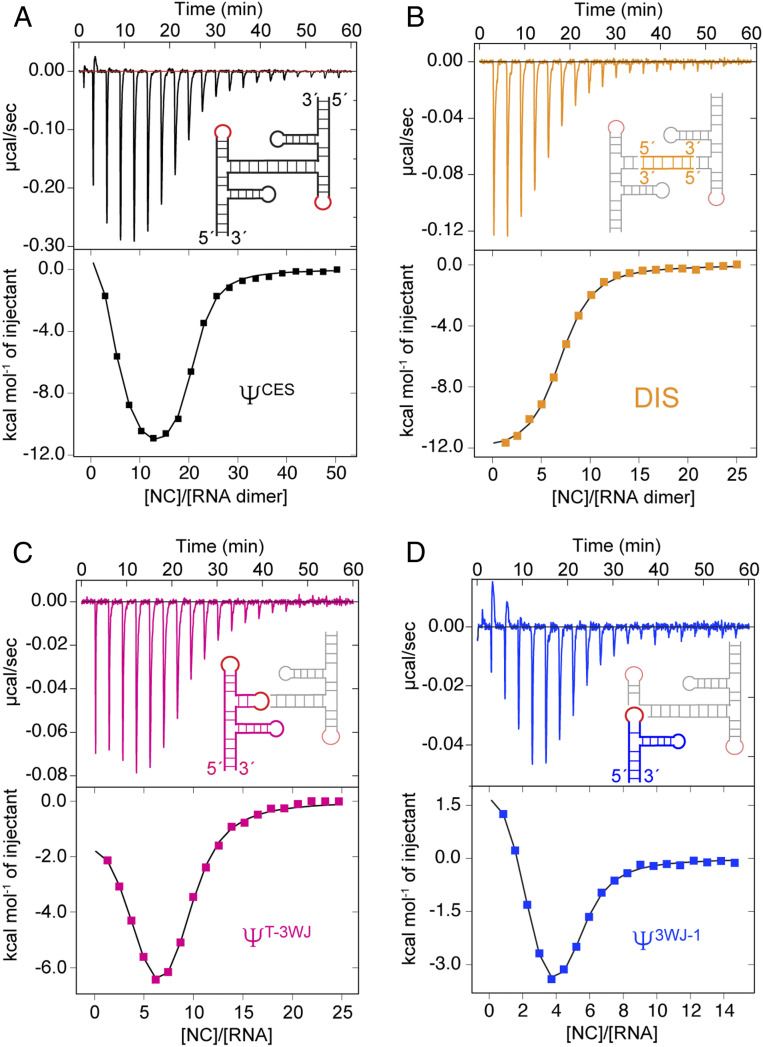
Localization of NC binding sites in [Ψ^CES^]_2_. Secondary structure models for [Ψ^CES^]_2_ (*A*) and its subdomains (*B*–*D*) are shown with ITC isotherms. Nonnative “GAGA” tetraloop is colored in red, the DIS region in yellow (*B*), Ψ^T−3WJ^ region in pink (*C*), and the Ψ^3WJ−1^ in blue (*D*).

The finding that the TAR-PolyA and PBS regions have very weak NC binding sites is consistent with the previous reports that Ψ^CES^, which lacks the TAR-PolyA and PBS elements, is the minimal region of the 5′-L required for packaging (30, 37). NC titrations with [Ψ^CES^]_2_ gave rise to a two-component ITC binding profile with quality sufficient for two-sets-of-sites model fitting (Fig. 2*A* and Table 1). The initial endothermic component of the isotherm corresponds to the binding of four NC proteins with average *K*_d_ values of 45 ± 19 nM, consistent with results obtained for the intact leader. Fits for the exothermic portion of the isotherm are indicative of ∼16 NC binding sites with moderate average affinities (*K*_d_ = 330 ± 20 nM), similar to the isotherms and affinities observed for the apical loop of the Ψ-hairpin (Table 1 and *SI Appendix*, Fig. S2*C*). These results indicate that the high-affinity (endothermic) binding sites reside within the Ψ^CES^ region of the 5′-L.

The [Ψ^CES^]_2_ structure comprises a central A-helical duplex that connects two tandem three-way junctions (30). To identify the locations of the NC binding sites within [Ψ^CES^]_2_, particularly the initial endothermic sites, we prepared three additional RNA constructs corresponding to the DIS duplex (residue 237 to 281 plus a nonnative G–C pair at each end of the duplex; *SI Appendix*, Fig. S1*F*) (46), the tandem three-way junction (all residues of Ψ^CES^ except the DIS helix, which was substituted by a “GAGA” tetraloop) (Ψ^T−3WJ^; *SI Appendix*, Fig. S1*E*), and the U5:AUG-H1-Ψ three-way junction (Ψ^3WJ−1^; *SI Appendix*, Fig. S1*G*). The NC binding stoichiometry and affinities of these [Ψ^CES^]_2_ subfragments were characterized by ITC (Fig. 2 *B*–*D*). The DIS dimer binds six NC molecules with exothermic energetics and affinity (average *K*_d_ = 340 ± 30 nM) similar to that observed for the apical loop of the Ψ-hairpin (Table 1). In contrast, the high-affinity endothermic phase of the NC binding energetics was retained by both the Ψ^T−3WJ^ and Ψ^3WJ−1^ constructs, indicating that the initial binding site resides within Ψ^3WJ−1^. Two-sets-of-sites fitting of the ITC data for Ψ^3WJ−1^ revealed that this fragment contains 2.2 ± 0.2 high-affinity endothermic NC binding sites (*K*_d_ = 28 ± 15 nM) and 2.7 ± 0.3 additional exothermic NC binding sites (*K*_d_ = 320 ± 180 nM). The exothermic binding sites across different subdomains of [Ψ^CES^]_2_ exhibit similar affinities with *K*_d_ in the range of 300 to 400 nM (Table 1). Thus, all of the high-affinity NC binding sites of the dimeric 5′-L are clustered within [Ψ^CES^]_2_, with the initial endothermic binding located within the Ψ^3WJ−1^ region of the RNA.

### NC Binds Initially to a Conserved [UUUU]:[GGAG] Element.

To identify the specific nucleotides that contribute to high-affinity endothermic binding, NMR-detected NC binding data were obtained for Ψ^3WJ−1^. The structure of the junction was previously characterized in the context of a larger RNA (155 nt) comprising half of the symmetric [Ψ^CES^]_2_ dimer (called Ψ^CESm^) (30). The improved NMR spectral quality obtainable for the 73-nt Ψ^3WJ−1^ construct enabled unambiguous assignment of nearly all of the nonexchangeable aromatic and ribose H1′ protons, facilitating structural and NC binding analyses.

**Fig. 3.**
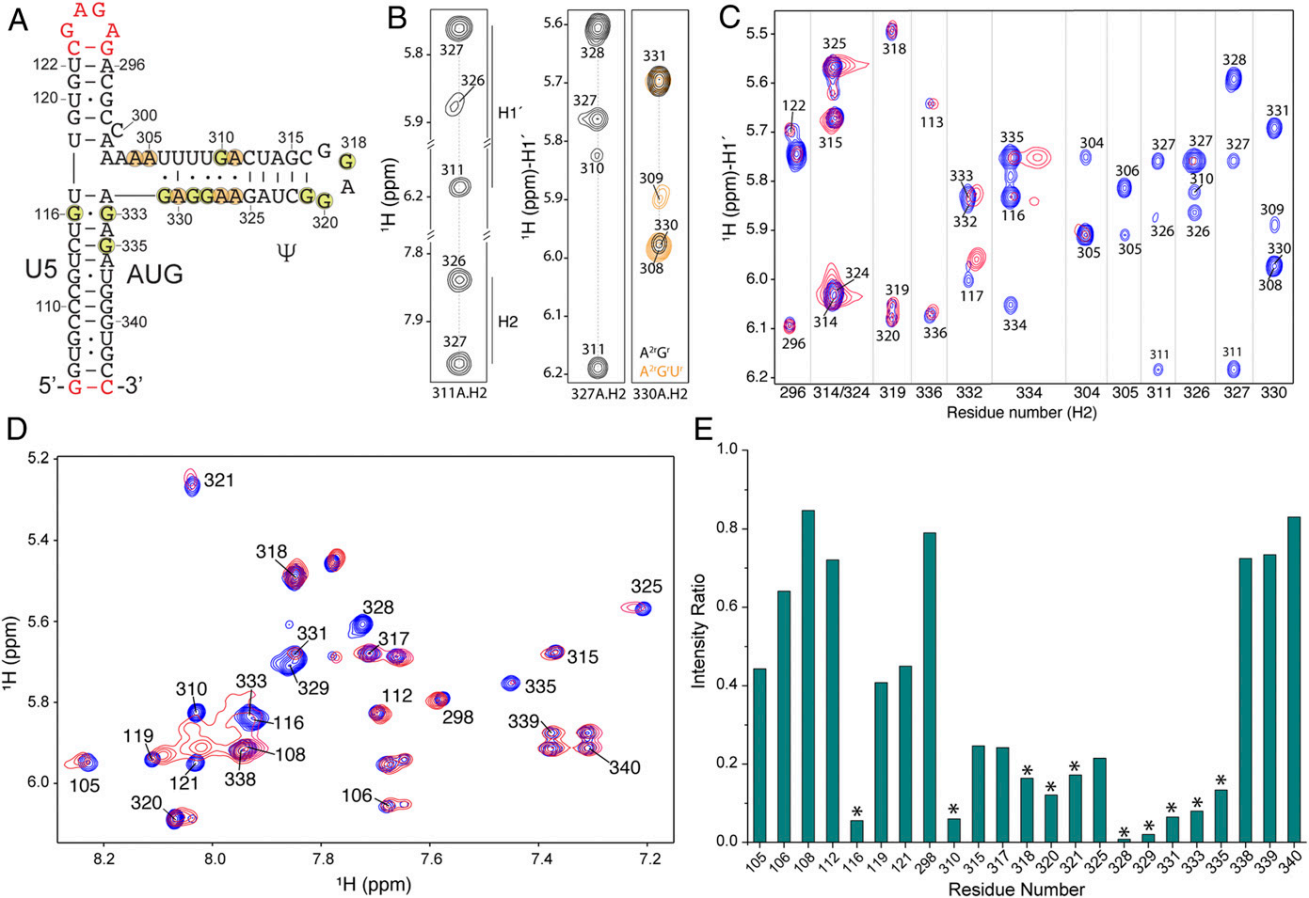
Structure and NC binding characterization of Ψ^3WJ−1^. (*A*) Secondary structure of Ψ^3WJ−1^. Nonnative residues are shown in red. Adenosines with chemical shifts significantly perturbed by NC binding are highlighted in orange, and guanosines in yellow. (*B*) Selected region of the 2D NOESY spectra of A^2r^G^r^– and A^2r^G^r^U^r^–Ψ^3WJ−1^. Cross-strand NOEs confirm the structure of the [UUUUAG]:[AAGGAG] region. (*C*) Two-dimensional NOESY spectra overlay of A^2r^G^r^U^r^–Ψ^3WJ−1^ with (red) and without (blue) twofold excess of NC. The H2–H1′ cross-peak region is shown. (*D*) Two-dimensional NOESY spectra overlay of G^H^–Ψ^3WJ−1^ with (red) and without (blue) twofold excess of NC. Residue numbers are shown for the H8–H1′ cross-peaks. (*E*) Intensity ratios of the protein-bound (red) and the free- (blue) RNA NMR signals in *D* are plotted against the residue number. Residues with severe signal intensity reduction are marked with asterisks.

All NMR signals from the fingerprint region of ^2^H-edited two-dimensional (2D) nuclear Overhauser effect spectroscopy (NOESY) spectra for A^H^-, G^H^-, C^H^-, or U^H^-labeled Ψ^3WJ−1^ RNAs (corresponding to RNAs with fully protonated adenosines, guanosines, cytosines, and uridines, respectively, with all other nucleotides in the sample perdeuterated) were assigned using standard methods (*SI Appendix*, Fig. S3) (47). ^2^H-edited 2D NOESY spectra were also obtained for A^2r^G^r^– and A^2r^G^r^U^r^–Ψ^3WJ−1^ samples (superscripts denote sites of protonation; e.g., A^2r^G^r^ corresponds to a sample with protonated adenosine-C2 and ribose carbons, and protonated guanosine ribose carbons, with all other nucleotides perdeuterated) (Fig. 3*B*). A311-H2 gave rise to a well-resolved NMR signal and exhibits cross-strand NOEs with the H2 and H1′ protons of both A326 and A327. Similar results were observed for A327-H2, which exhibits cross-strand NOEs with the H1′ protons of G310 and A311, providing evidence for the formation of noncanonical G310:A327 and A311:A326 base pairs. The cross-strand NOEs between A330-H2 and U308-H1′, as well as A330-H2 and U309-H1′, indicate that the “UUUU” and “GGAG” sequences are close in 3D structure (the two H2–H1′ proton pairs separated by <5.5 Å) and likely form a [UUUU]:[GGAG] duplex with multiple G:U wobble base pairs (Fig. 3*A*). However, the weaker cross-strand NOE between A330-H2 and U308-H1′, in comparison with the sequential A330-H2 to G331-H1′ NOE, suggests a dynamic nature of the interstrand interactions of the [UUUU]:[GGAG] region (Fig. 3*B*). The structure differs from one proposed in a previous NMR study in which the residues of the [UUUU]:[GGAG] stem were proposed to be nonbase paired and partially disordered (48), possibly due to differences in the constructs or experimental conditions employed, but is consistent with NMR findings for a larger 5′-L fragment (30).

NMR titration experiments were subsequently performed to identify the nucleotides directly involved in NC binding. Surprisingly, adenosine-H2 signals of residues A304, A305, A311, A326, A327, and A330, which reside within or near the [UUUU]:[GGAG] helix, were all substantially perturbed when a twofold excess of NC was added, whereas signals of A314, A319, and A324 from the well-known NC binding Ψ-apical loop were barely affected (Fig. 3 *A* and *C*). Since HIV-1 NC binds with high affinity to unpaired or weakly paired guanosines (39), we also performed NC titration experiment with a G^H^-labeled Ψ^3WJ−1^ sample (Fig. 3 *D* and *E*). Consistent with findings from the adenosine-H2 detected titrations, signals for G328 and G329 of the [UUUU]:[GGAG] duplex were significantly broadened, whereas the guanosines of the Ψ-apical loop were relatively unaffected (Fig. 3 *D* and *E*). Thus, these data provide direct evidence that binding of NC to the guanosines of the dynamic [UUUU]:[GGAG] region are responsible for the initial high-affinity endothermic binding.

### [UUUU]:[GGAG] Lability Is Required for High-Affinity NC Binding.

**Fig. 4.**
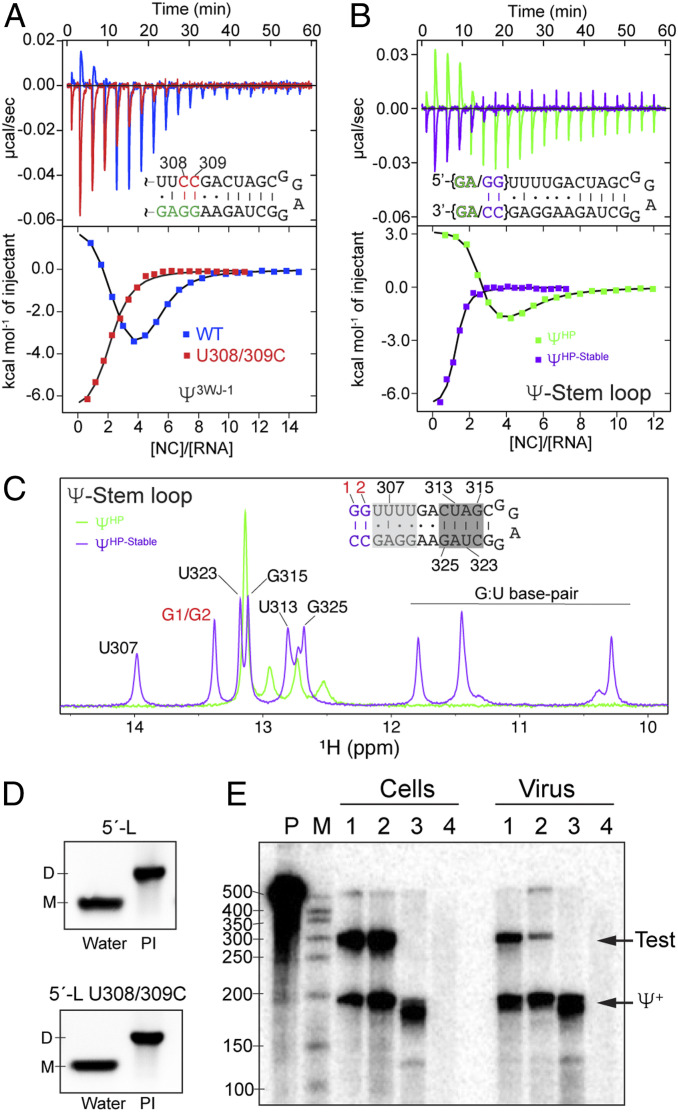
Stabilizing the stem region of Ψ-hairpin eliminates high-affinity endothermic binding and impairs in vivo genome packaging. (*A*) ITC data for wild-type Ψ^3WJ−1^ (blue) and the U308/309C mutant (red). (*B*) ITC data for Ψ^HP-stable^ (purple) and Ψ^HP^ (green). (*C*) Overlay of 1D imino proton spectra for Ψ^HP-stable^ (purple) and Ψ^HP^ (green). Imino proton chemical shifts assignments are shown for Ψ^HP-stable^. (*D*) Dimerization gel shift for wild-type 5′-L and the U308/309C mutant. (*E*) The G:U to G–C mutations significantly reduced the RNA packaging in competition with the wild-type RNA. Lanes 1 and 2 are native HIV-1 5′-L versus test vectors containing wild-type 5′-L and G:U to G–C mutant, respectively. Lane 3 is HIV-1 5′-L helper expressed without test RNA. Lane 4 is mock transfected cells.

We next asked whether the endothermic nature of the binding is due to the disruption of the G:U wobble base pairs in the [UUUU]:[GGAG] helix. To stabilize the stem, two of the uridines within this helix (U308 and U309) were substituted by cytosines, thereby converting two G:U base pairs in Ψ^3WJ−1^ to G–C pairs. Interestingly, these substitutions completely eliminated high-affinity endothermic NC binding to Ψ^3WJ−1^ (Fig. 4*A*). These findings suggest that structural lability of the [UUUU]:[GGAG] helix may be important for high-affinity endothermic NC binding.

To further investigate structural and NC binding features of the [UUUU]:[GGAG] helix, two Ψ-hairpin RNAs that extend through the [UUUU]:[GGAG] element were prepared, one with 5′- and 3′-terminating residues that cannot form Watson–Crick base pairs (5′-GA and AG-3′, respectively; Ψ^HP^) (*SI Appendix*, Fig. S1*H*), and the other with helix-stabilizing 5′-GG and CC-3′ terminating residues at the 5′- and 3′-ends, respectively (Ψ^HP-stable^) (Fig. 4*C* and *SI Appendix*, Fig. S1*I*). NMR chemical shifts and cross-peak patterns observed for the nonstabilized Ψ^HP^ construct were similar to those of corresponding residues in Ψ^3WJ−1^ (*SI Appendix*, Figs. S4 and S5), indicating that the structures are similar. Spectra for both constructs exhibited sequential H8/H6-to-H1′ and cross-strand adenosine-H2 to H1′ NOEs for residues 326-AAGGAG-331 and 306-UUUUGA-311 (spectrum for Ψ^HP^ shown in *SI Appendix*, Fig. S4) consistent with a helical structure. However, Watson–Crick imino signals for the [UUUU]:[GGAG] element were broadened beyond detection in ^1^H NMR data obtained for Ψ^HP^ but were readily observable in spectra obtained for Ψ^HP-stable^ (Fig. 4*C*), indicating that the [UUUU]:[GGAG] element of Ψ^HP^ is structurally labile. Importantly, as observed for Ψ^3WJ−1^, NC titrations with Ψ^HP^ afforded two-component ITC isotherms with initial high-affinity endothermic binding (*n* = 2.3 ± 0.2; *K*_d_ = 26 ± 21 nM) followed by exothermic binding (*n* = 2.8 ± 0.3; *K*_d_ = 1.5 ± 0.2 µM), whereas the RNA containing helix-stabilizing G–C closing base pairs (Ψ^HP-stable^) exhibited only exothermic NC binding behavior (*n* = 1.2 ± 0.3; *K*_d_ = 300 ± 150 nM) (Fig. 4*B* and Table 1). These findings further indicate that high-affinity endothermic NC binding to [UUUU]:[GGAG] occurs with concomitant unfolding and/or structural rearrangement of this element. Notably, a modified Ψ^HP^ construct that lacks the “UUUU” nucleotides and leaves the GGAG-3′ element unpaired did not bind NC with the same high affinity as observed for the intact RNA (*SI Appendix*, Fig. S2*D*). Moreover, the Ψ-apical loop and the [UUUU]:[GGAG] element both contain an identical “GGAG” sequence but exhibit distinct binding energetics and affinities (Table 1). Thus, it appears that the lability and the structure of the [UUUU]:[GGAG] element are both required for high-affinity NC binding.

### [UUUU]:[GGAG] Element Promotes Efficient HIV-1 RNA Packaging.

To investigate whether the [UUUU]:[GGAG] element is important for HIV-1 genome packaging, we compared the packaging efficiencies of vector RNAs containing the wild-type and U308/309C mutated leaders. The vector with the wild-type leader (L688-RRE-Puro-LTR) was previously shown to display competitive RNA packaging levels indistinguishable from those of HIV-1 gRNAs (37). The U308/309C mutations did not affect the dimerization property of the 5′-L in vitro (Fig. 4*D*), as expected since they also did not significantly perturb the structures of well-characterized 5′-L fragments (see above and *SI Appendix*, Fig. S6). However, the packaging efficiency of the U308/309C mutant was substantially diminished compared to that of the wild-type vector (13 ± 2%) (Fig. 4*E*). Thus, the U308/309C double mutation that stabilizes the [UUUU]:[GGAG] element and prevents high-affinity endothermic NC binding in vitro profoundly impairs HIV-1 RNA packaging in transfected cells.

### An HIV-1 RNA Packaging Inhibitor Targets the [UUUU]:[GGAG] Element.

**Fig. 5.**
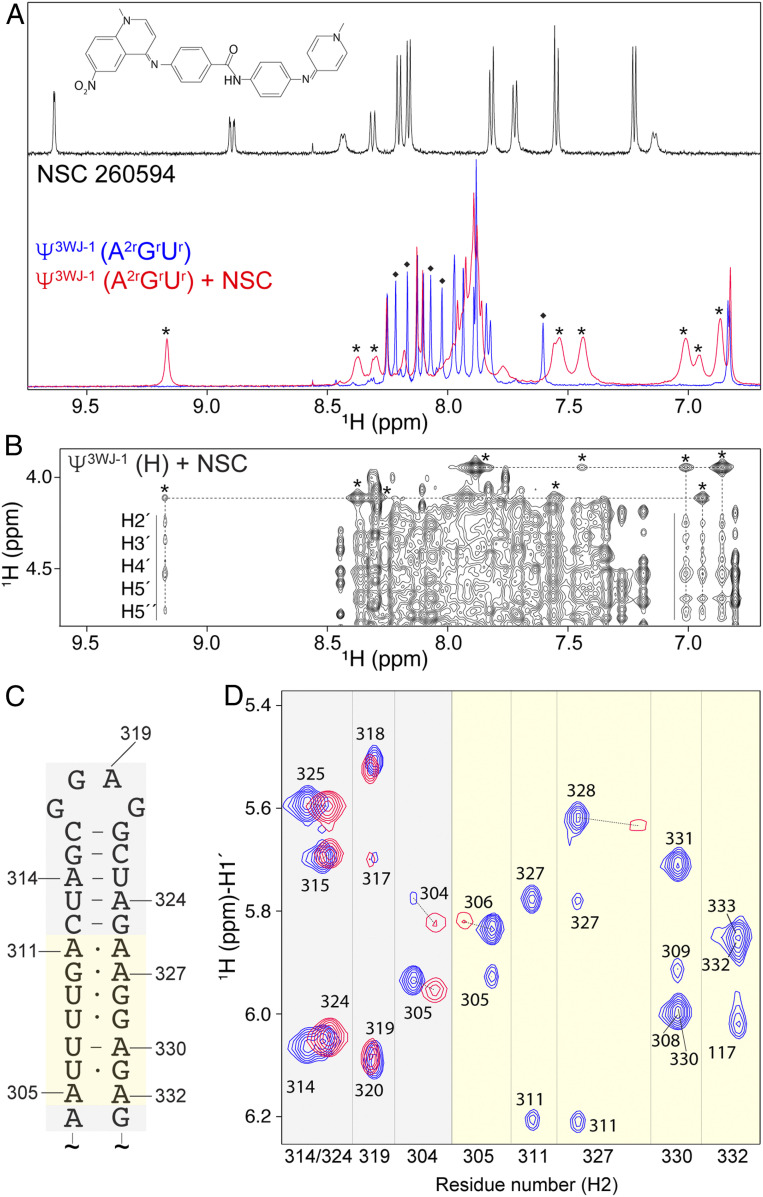
NSC 260594 binds to the stem region of Ψ-stem loop. (*A*) One-dimensional ^1^H spectrum for NSC (*Top*). Overly of 1D ^1^H spectra of A^2r^G^r^U^r^–Ψ^3WJ−1^ with (red) or without (blue) twofold excess of NSC (*Bottom*). The ^1^H signals of the NSC compound in the RNA-bound state are indicated by asterisks. The ^1^H signals of the Ψ^3WJ−1^ with severe line broadening after NSC binding are indicated by diamonds. (*B*) NOESY spectrum of fully protonated Ψ^3WJ−1^ with twofold excess of NSC. Asterisks denote intramolecular NOEs between aromatic and methyl protons of NSC. Intermolecular NOEs between NSC aromatic protons and RNA ribose protons are marked by vertical lines. (*C*) Secondary structure of the Ψ-stem region. (*D*) Two-dimensional NOESY overlay of A^2r^G^r^U^r^–Ψ^3WJ−1^ with (red) or without (blue) twofold excess of NSC. Light yellow denotes the signals that are severely perturbed by NSC binding. Light gray shows the residues that are mildly affected by NSC binding.

The requirement of the [UUUU]:[GGAG] element for high-affinity NC binding and RNA packaging suggested that this region of the leader might serve as a potent HIV-1 therapeutic target. Recently, a small-molecule quinolinium derivative (NSC 260594; hereafter referred to as NSC) was shown to inhibit HIV-1 RNA packaging, apparently by binding to the Ψ-hairpin region of the HIV-1 5′-L and exerting a global stabilizing effect on the RNA structure (49). NSC was originally identified in a screen for small molecules that inhibit Gag binding to the apical “GGAG” loop of the Ψ-hairpin (50). We extended those studies to characterization of NSC interactions with the larger Ψ^3WJ−1^ RNA using ^2^H-edited NMR. Many ^1^H NMR signals of NSC exhibited substantial chemical shifts (and some line broadening) when bound to a A^2r^G^r^U^r^-labeled Ψ^3WJ−1^ sample (compare Fig. 5 *A*, *Top*, and Fig. 5 *A*, *Bottom*), suggesting specific interactions between the compound and RNA. Intermolecular NOEs between the RNA ribose protons and the compound were also distinguishable (Fig. 5*B*). Several adenosine-H2 signals of Ψ^3WJ−1^ were significantly perturbed and exhibited reduced intensities (Fig. 5*A*). Two-dimensional NOESY data obtained for A^2r^G^r^U^r^-labeled Ψ^3WJ−1^ revealed that H2 signals of adenosines near the apical loop of the Ψ-hairpin (A314, A319, and A324) were barely perturbed upon NSC binding, whereas the H2 signals of adenosines near stem region of the Ψ-hairpin exhibited substantial broadening (Fig. 5 *C* and *D*). These findings indicate that the NSC RNA packaging inhibitor targets residues of the [UUUU]:[GGAG] element of the HIV-1 packaging signal and not the apical loop of the Ψ-hairpin.

## Conclusions

Early efforts to identify HIV-1 RNA packaging determinants identified stretches of nucleotides surrounding (and including) the Ψ-hairpin that, if deleted, resulted in severe HIV-1 packaging defects (23–25), and this led to proposals that the Ψ-hairpin is a major RNA packaging determinant. Studies suggested that the apical tetraloop of the Ψ-hairpin was the critical Gag/NC recognition element (48, 51), and its complex structure with NC revealed that two exposed guanosines from the “GGAG” loop are inserted into hydrophobic clefts on the surfaces of the two zinc finger domains of NC (39). Subsequent studies showed that substitution of the GGAG loop by GCUA abolished NC binding but did not significantly affect RNA packaging (16, 52), and it soon became clear that regions other than the apical loop of the Ψ-hairpin, including sequences in the stem of that hairpin (52, 53), are important for competitive RNA packaging (6, 26, 37, 54).

The present findings reveal that the conserved [UUUU]:[GGAG] element located at the base of the Ψ-hairpin and adjacent to a tandem three-way junction structure is critical both for high-affinity NC binding and competitive RNA packaging. Each of these two elements in the dimeric 5′-L binds two NC molecules with affinities (∼40 nM) approximately an order of magnitude greater than those of other NC binding sites within the encapsidation-promoting region of the viral leader, and more than 30 times tighter than NC binding sites in other regions of the leader (Table 1). The absence of this [UUUU]:[GGAG] element from spliced viral transcripts may contribute to the selective packaging of the full-length gRNA. The [UUUU]:[GGAG] element adopts a labile helical structure in the absence of NC and undergoes structural changes, possibly helix melting, upon endothermic binding by NC. These findings appear consistent with chemical probing studies showing that residues of [UUUU]:[GGAG] are resistant to RNase V1 cleavage in the absence of NC but exhibit enhanced chemical accessibility after Gag/NC binding (48, 55).

High-affinity NC binding sites identified in the RNA packaging signals of other retroviruses also appear to be structurally dynamic (56–60). For example, the Moloney murine leukemia virus (MoMuLV) NC binds specifically to a “UCUG” single-stranded region that links two stem loops (DIS-2 and SL-C of the MoMuLV packaging signal) with *K*_d_ ∼100 nM (56, 57). The Rous sarcoma virus (RSV) packaging signal is a comparatively small four-way junction (82-nt) with three stem loops (SLA, SLB, and SLC) and the O3 stem. The high-affinity NC recognition element of RSV (*K*_d_ ∼2 nM at low salt condition) is a two-component RNA structure, with the “UGCG” tetraloop of SLC bound by the N-terminal zinc finger of NC and the “AUG” residues linking SLA and SLB recognized by the C-terminal zinc finger (58, 59). The present studies show that structurally labile [UUUU]:[GGAG] elements serve as the initial sites for Gag binding to the HIV-1 leader. Thus, a general mechanism may be employed by retroviruses in which genome packaging is initiated by the binding of a small number of Gag molecules to a cluster of exposed and/or labile high-affinity NC binding sites, which stimulates recruitment of additional Gag molecules and nucleates virus assembly.

Our findings also indicate that the previously characterized HIV-1 RNA packaging inhibitor NSC binds to the [UUUU]:[GGAG] element of the Ψ^3WJ−1^ RNA. The fact that both NSC and NC bind tightly to the apical loop of Ψ-hairpin constructs that lack the [UUUU]:[GGAG] element and shift their binding preferences to the [UUUU]:[GGAG] helix in larger RNA constructs (50) suggests that the “GGAG” sequence (also present in the apical loop of the Ψ-hairpin) is important for binding but insufficient for high-affinity binding. The potent inhibitory effect of NSC on RNA packaging (49), and the sensitivity of helix stabilizing (and NC-blocking) G:U to G–C mutations, are mutually consistent with the proposal that high-affinity NC binding to the [UUUU]:[GGAG] site is essential for genome packaging. Our findings are also consistent with chemical probing analyses, suggesting that the inhibitory function of NSC is due to its stabilization of the Ψ-hairpin region of the leader (49). These collective findings suggest that the HIV-1 packaging signal, and particularly the [UUUU]:[GGAG] helix that bridges the Ψ-hairpin and a three-way junction structure, could serve as a potent RNA target for HIV therapeutic development.

## Materials and Methods

### Preparation of DNA Templates.

The DNA templates for the transcription of DIS and Ψ-hairpin derivative RNAs were prepared by annealing a 17-nt T7 promoter sequence (Top17, 5′-TAA TAC GAC TCA CTA TA-3′) to the corresponding reverse oligonucleotides (IDT): DIS, 5′-mGmGC CCC TCG CCT CTT GCC GTG CGC GCT TCA GCA AGC CGA GTC CTG CCT ATA GTG AGT CGT ATT A-3′; Ψ^HP^, 5′-mCTC TCC TTC TAG CCT CCG CTA GTC AAA ATC TAT AGT GAG TCG TAT TA-3′; Ψ^HP-stable^, 5′-mGmGC TCC TTC TAG CCT CCG CTA GTC AAA ACC TAT AGT GAG TCG TAT TA-3′; Ψ-apical loop, 5′-mGmGA CTA GCC TCC GCT AGT CCT ATA GTG AGT CGT ATT A-3′; Ψ-fragment, 5′-mCTC CTT CTA GCC TCC GCT AGT CTA TAG TGA GTC GTA TTA (m denotes 2′-*O*-methyl modification to reduce nontemplated nucleotide addition by T7 RNA polymerase) (61). Briefly, Top 17 (20 µL, 600 µM) was mixed with reverse DNA oligo (40 µL, 200 µM). The mixture was then boiled for 3 min and slow cooled overnight. H_2_O (940 μL) was added to yield 1-mL DNA template, which was directly used in the transcription reaction.

The DNA templates for other larger RNA constructs were obtained by standard PCR amplification (EconoTaq PLUS 2× Master Mix; Lucigen) using pUC19 plasmids containing the Top17 sequence and the RNA encoding sequences. A common forward amplification primer 80 nt upstream to the T7 promoter sequence was used for all constructs (5′-GGG ATG TGC TGC AAG GCG ATT AAG TTG GG-3′). The reverse amplification primers are 5′-mCmGC ACC CAT CTC TCT CCT TCT AGC CTC C-3′ for 5′-L, 5′-L U308/309C, and 5′-L^ΔPBS^; 5′-mGmGC ACC CAT CTC TCT CCT TCT AGC CTC C-3′ for Ψ^CES^, Ψ^T−3WJ^, Ψ^3WJ−1^, and Ψ^3WJ−1^ U308/309C; 5′-mCmAC TAC TTT GAG CAC TCA AGG CAA GC-3′ for TAR-PolyA.

The construction of plasmids for 5′-L, 5′-L^ΔPBS^, and Ψ^CES^ were described previously (30, 37). The DNA fragment for Ψ^T−3WJ^ was generated by overlapping PCR using the Ψ^CES^ plasmid as the template with the primers 5′-CCT AGT AAG GAA TTC TAA TAC GAC TCA CTA TAG GTG CCC GTC TG-3′ and 5′-GTA CTC ACC AGT CGT CTC CGA GAG ATC TCC TC-3′ for the first fragment, and primers 5′-GAG GAG ATC TCT CGG AGA CGA CTG GTG AGT AC-3′ and 5′-CTT ACT AGG GGA TCC CCC GGG CAC-3′ for the second fragment. The amplified full-length DNA fragment for Ψ^T−3WJ^ was ligated into the pUC19 plasmid after digestion with EcoRI and BamHI (New England Biolabs). The plasmid for Ψ^3WJ−1^ was generated using the same overlapping PCR method with 5′-CTG TTG TGT CGA GAG ACG CCA AAA ATT TTG AC-3′ and 5′-TTT TGG CGT CTC TCG ACA CAA CAG ACG-3′ as the two overlapping primers. Plasmid for 5′-L U308/309C was prepared by site-directed mutagenesis (QuikChange Lightning; Agilent) on 5′-L plasmid using forward primer 5′-GGT GAG TAC GCC AAA AAT TCC GAC TAG CGG AGG CTA GAA GG-3′ and reverse primer 5′-CCT TCT AGC CTC CGC TAG TCG GAA TTT TTG GCG TAC TCA CC-3′. Plasmid for Ψ^3WJ−1^ U308/309C was prepared similarly using Ψ^3WJ−1^ plasmid and forward primer 5′-GTC GAG AGA CGC CAA AAA TTC CGA CTA GCG GAG GCT AGA AGG-3′ and reverse primer 5′-CCT TCT AGC CTC CGC TAG TCG GAA TTT TTG GCG TCT CTC GAC-3′.

### RNA In Vitro Transcription.

RNAs were prepared by in vitro transcription using T7 RNA polymerase (purified in-house) in 7.5- to 30-mL reactions. A 7.5-mL reaction contained ∼0.25 mg of PCR-amplified DNA template or ∼4 nmol of annealed DNA template, 20 mM MgCl_2_, 3 to 6 mM NTPs, 2 mM spermidine, 2 mM DTT, 20% (vol/vol) DMSO, 80 mM Tris⋅HCl (pH 9.0), and 0.15 mg of T7 RNA polymerase. The reaction was quenched after a 5-h incubation at 37 °C by addition of an EDTA-urea mixture (250 mM EDTA, 7 M urea, pH 8.0). The reaction mixture was boiled for 5 min and snap cooled on ice for 5 min prior to addition of glycerol (final concentration, 6% [vol/vol]). RNAs were purified by electrophoresis on urea-containing polyacrylamide denaturing gels (SequaGel; National Diagnostics) at 20 W overnight, visualized by UV shadowing, and eluted using the Elutrap electroelution system (Whatman) at 150 V overnight. The eluted RNAs were concentrated and washed twice with 2 M high-purity NaCl followed by extensive desalting using Amicon Ultra Centrifugal Filter Device (Millipore).

### Preparation of NTPs.

Perdeuterated (A^D^, G^D^, C^D^, and U^D^) and partially deuterated (U^r^) NTP reagents used for in vitro transcription were purchased from Cambridge Isotope Laboratories (CIL). A^2r^ and G^r^ were obtained by deuteration of the C8 position of fully protonated ATP and GTP. A volume of 140 µL of triethylamine (TEA) was added to 0.2 g of ATP or GTP dissolved in 8 mL of 99.8% D_2_O (CIL). One-milliliter aliquots of ATP and GTP were incubated at 60 °C for 5 d and 24 h, respectively. TEA was subsequently removed by lyophilization.

### NC Purification.

The pET-3a plasmid containing the DNA fragment encoding HIV-1_NL4-3_ NC protein (55 amino acids with the sequence of MQKGN FRNQR KTVKC FNCGK EGHIA KNCRA PRKKG CWKCG KEGHQ MKDCT ERQAN) was transformed into BL21 (DE3) pLysE. The protein was overexpressed and purified as described previously (37). Briefly, the cells were lysed by freeze-thawing and microfluidization. After centrifugation, the supernatant containing NC protein was applied to ion exchange chromatography using tandem Q- and SP- columns (the Q-column was removed before elution). Protein was further purified with size exclusion chromatography on a Superdex 30 column in PI buffer containing 5 mM TCEP.

### ITC.

ITC experiments were carried out using an ITC200 (MicroCal Corporation). A volume of 40 µL of NC (120 to 240 μM) in ITC buffer (10 mM Tris⋅HCl, pH 7.0, 140 mM KCl, 10 mM NaCl, 5 mM MgCl_2_, and 5 mM TCEP) was loaded into the injection syringe. The calorimetry cell was loaded with 200 µL of RNA (0.6 to 4 μM) in the same buffer as NC. After thermal equilibration at 30 °C and the initial 60-s delay, a single injection of 0.2 μL followed by 19 serial injections of 2 μL were made into the calorimetry cell.

### Native Gel Electrophoresis.

RNA (1 µM) was prepared in PI buffer with 10 mM Tris⋅HCl (pH 7.5), 140 mM KCl, 10 mM NaCl, and 1 mM MgCl_2_, heat denatured for 3 min, and slowly cooled down to room temperature. The 500-ng RNA samples were loaded onto 1% agarose gels prestained with ethidium bromide and run at 110 V in 1× TB buffer (44.5 mM Tris–boric acid, pH 7.5) on ice to prevent thermal denaturation during electrophoresis.

### NMR Spectroscopy.

All NMR data were collected with 600- or 800-MHz Bruker AVANCE spectrometer equipped with cryoprobe. NMR samples were prepared in 10 mM Tris-d11 buffer with an RNA concentration of ∼200 µM in a standard 5-mm NMR tube (500 µL). Nonexchangeable ^1^H assignments were obtained from 2D NOESY experiments (NOE mixing time, 300 ms; relaxation delay, 3.2 s; *T* = 35 °C) using samples in D_2_O (99.8%; CIL). Imino ^1^H assignments were obtained from 2D NOESY experiments with jump-return water suppression (*T* = 10 °C) using samples in 90% H_2_O/10% D_2_O. The NSC compound (provided by National Cancer Institute–Developmental Therapeutics Program; https://dtp.cancer.gov/) used for NMR titration experiments was dissolved in DMSO-d6 (CIL) with a final concentration of 20 mM and stored at −20 °C. All NMR data were processed with NMRFx (62) and analyzed with NMRViewJ (63).

### In Vivo Packaging Experiments.

The Ψ^+^ helper NL4-3 GPP contains HIV-1 NL4-3 sequences with *env* replaced by a puromycin-resistance expression cassette and has been described previously (29). The test vector L688-RRE-Puro-LTR contains 688 base pairs of the wild-type HIV-1 NL4-3 leader and has been described previously (37). U308/309C mutation was introduced into L688-RRE-Puro-LTR by replacement of the corresponding region in the test vector with a fragment generated using overlap extension PCR.

Human 293T cells were cultured at 37 °C under 5% CO_2_ in DMEM media, containing 10% FBS and 50 µg/mL gentamicin. To generate virions for RNA packaging analysis, 293T cells at 70% confluence in 10-cm plates were cotransfected with a total of 6 μg of plasmid DNA that included one test vector plasmid and NL4-3 GPP at an ∼1:1 molar ratio using polyethylenimine (64). Cells and media were harvested 48 h after transfection. Media was filtered through 0.22-μm filters and viral particles concentrated by ultra-centrifugation (25,000 rpm) through a 20% sucrose cushion. RNA from virus pellet and cells was isolated using TRIzol according to the manufacturer’s protocol (Ambion) and DNase treated.

RNA content of transfected cells and viral particles was analyzed by RNase protection assay (RPA) as previously described (65). The chimeric riboprobe HIVgag/CMV used in this study was designed to protect a 201-nt fragment of HIV-1 gag sequence (presented only in NL4-3 GPP helper) and 289 nt of CMV promoter sequence (presented only in test vectors). Riboprobe was transcribed from linearized plasmid templates using T7 RNA polymerase (Promega) and [α-^32^P]-rCTP (Perkin-Elmer). Products of RPA were quantified by phosphorimaging using standard methods.

## Supplementary Material

Supplementary File

## Data Availability

All necessary data for substantiating the conclusions in the manuscript are included in the manuscript and *SI Appendix*. Additional data are available upon request from the authors.
