# Supplementary material for: Identification of the initial nucleocapsid recognition element in the HIV-1 RNA packaging signal
Source: Proc Natl Acad Sci U S A. 2020 Jul 9;117(30):17737–46. doi: 10.1073/pnas.2008519117 (PMC7395439; doi:10.1073/pnas.2008519117)
Supplement: Supplementary File [file pnas.2008519117.sapp.pdf]

SUPPLEMENTARY MATERIALS

Identification of the initial nucleocapsid recognition element in the HIV-1 RNA packaging signal

Pengfei Ding[a,b], Siarhei Kharytonchyk[c], Alexis Waller[a], Ugonna Mbaekwe[a], Sapna Basappa[a], Nansen Kuo[a], Heather M. Frank[a], Christina Quasney[a], Aaron Kidane[a], Canessa Swanson[a], Verna Van[a], Mitali Sarkar[a], Emily Cannistraci[a], Ridhi Chaudhary[a], Hana Flores[a], Alice Telesnitsky[c,1], Michael F. Summers[a,b,1]

[a]Department of Chemistry and Biochemistry, University of Maryland Baltimore County, Baltimore, MD 21250;

[b]Howard Hughes Medical Institute, University of Maryland Baltimore County, Baltimore, MD 21250;

[c]Department of Microbiology and Immunology, University of Michigan Medical School, Ann Arbor, MI 48109

[1]To whom correspondence may be addressed. Email: summers@hhmi.umbc.edu or ateles@umich.edu

CONTENTS:

CONTAINS SIX FIGURES AND FIGURE CAPTIONS

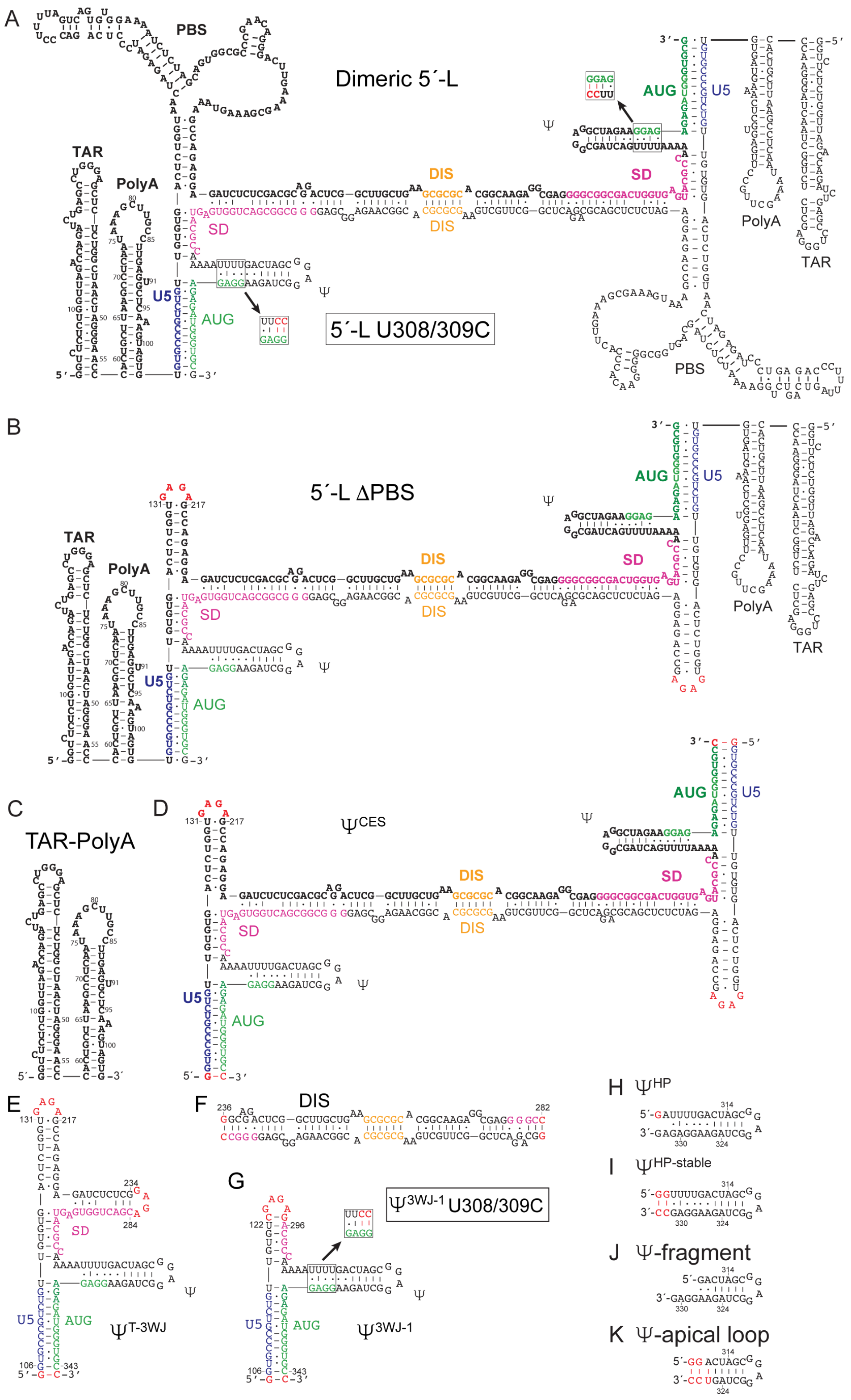

Fig. S1. Summary of all RNA constructs used in this study. Non-native residues are shown in red.

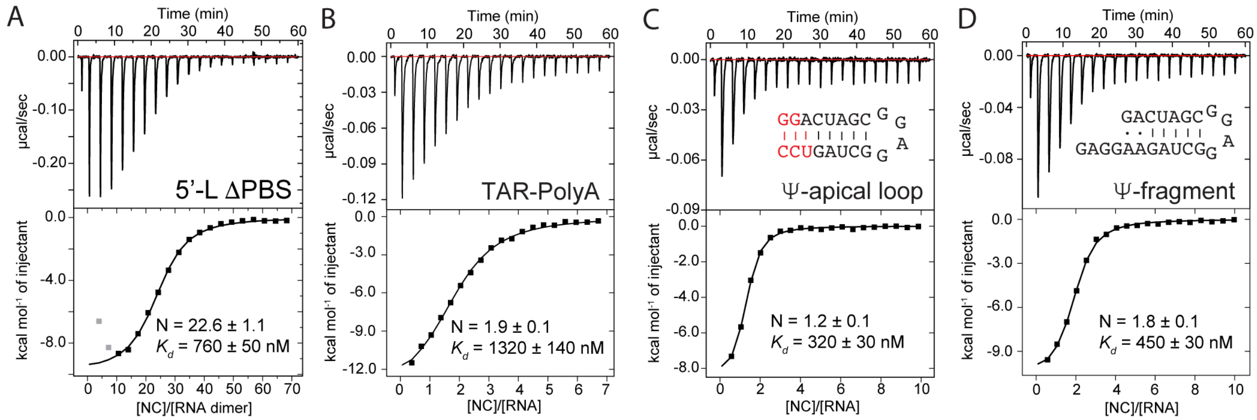

Fig. S2. ITC data of HIV-1 5´-L constructs. (*A*) 5´-L with the PBS region deleted. The gray data points due to endothermic binding were not included in data fitting. (*B*) TAR and PolyA hairpins; (*C*) the upper stem region and the apical loop from the Ψ-stem loop; (*D*) A construct with Ψ-apical loop and a single stranded "GGAG" region.

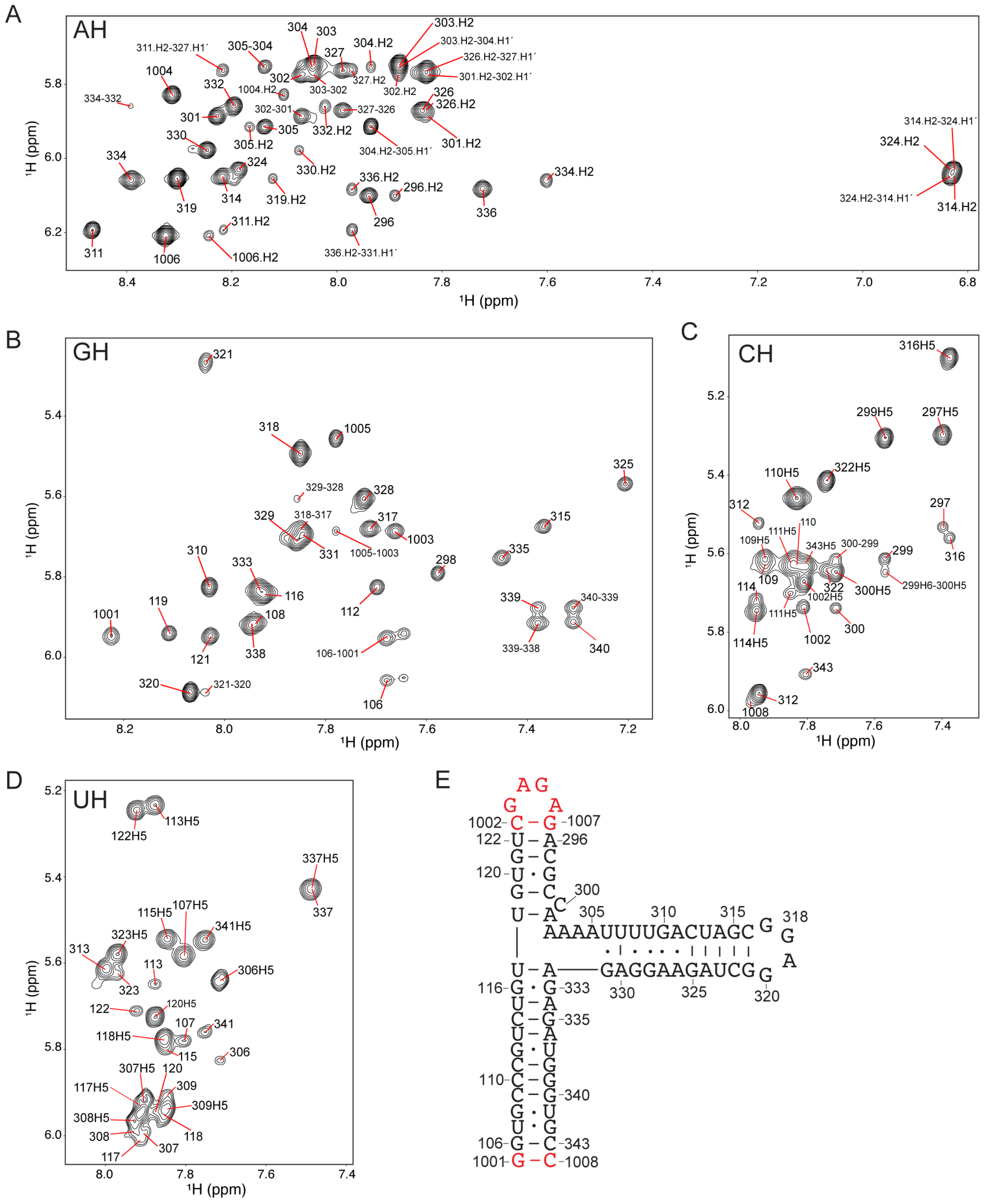

Fig. S3. Chemical shift assignments for Ψ^{3WJ-1}. For A^{H}- (*A*) and G^{H}- (*B*) spectra, intra-residue H8-H1´ cross-peaks are denoted by residue number; inter-residue H8-H1´ are indicated by (residue number of H8)-(residue number of H1´); Intra-residue H2-H1´ are

shown by (residue number).H2; Inter-residue H2-H1´ are labeled by (residue number of H2).H2-(residue number of H1´).H1´. For C^H- (*C*) and U^H- (*D*) spectra, intra-residue H6-H1´ cross-peaks are denoted by residue number; inter-residue H6-H1´ are indicated by (residue number of H6)-(residue number of H1´); Intra-residue H6-H5 cross-peaks are labeled by (residue number).H5; Inter-residue H6-H5 cross-peaks are indicated by (residue number of H6).H6-(residue number of H5).H5. (*E*) Secondary structure of Ψ^{3WJ-1}. Non-native residues are shown in red and numbered 1001-1008.

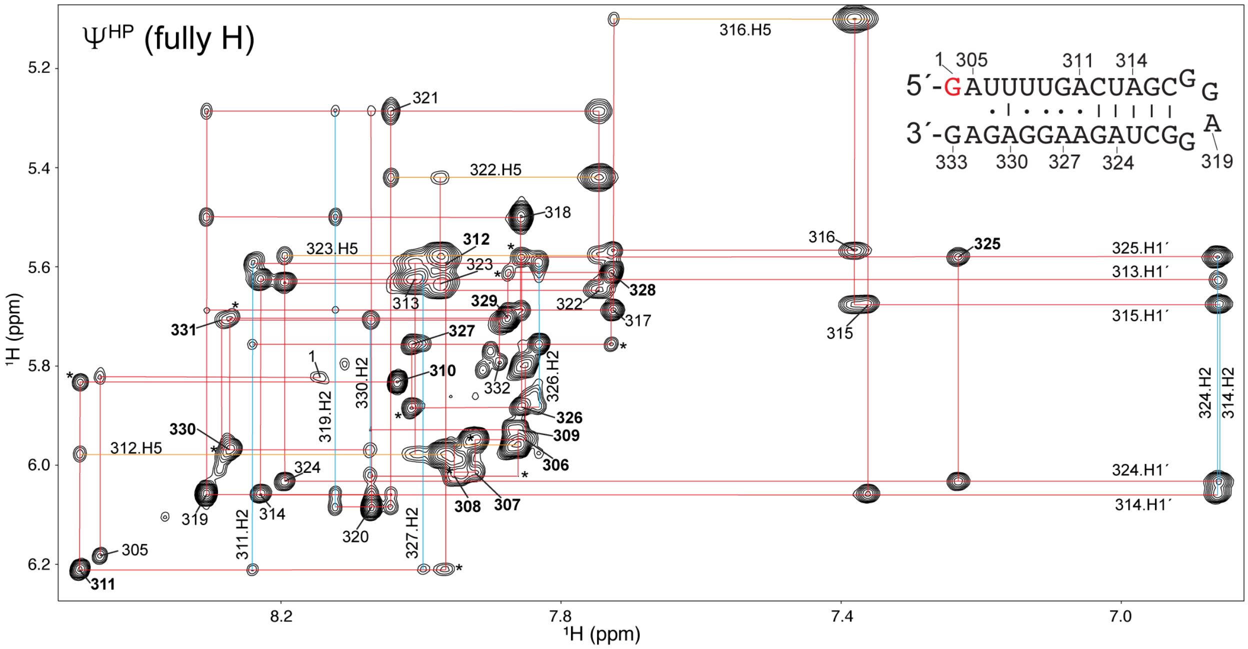

Fig. S4. Chemical shift assignments for Ψ^{HP}. Intra-residue H8/H6-H1´ cross-peaks are denoted by residue number. Adenosine H2 signals are indicated by blue lines; Pyrimidine H5 signals are labeled by yellow lines. Sequential H8/H6-H1´ cross-peaks for the [UUUUGAC]:[GAAGGAG] region are marked by stars.

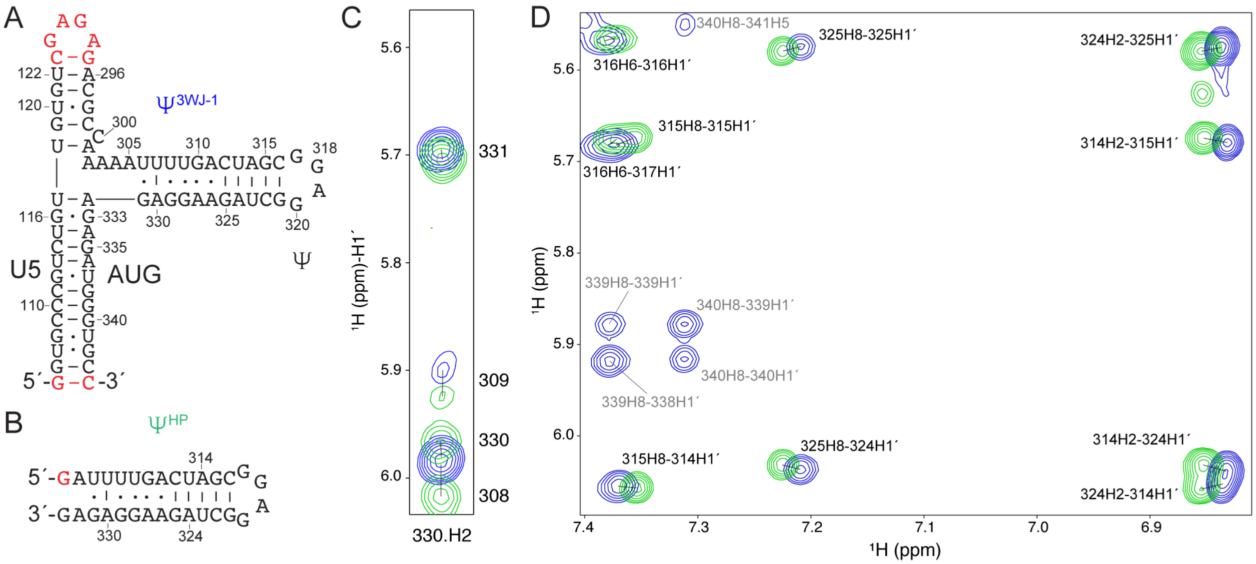

Fig. S5. Ψ^{HP} is structurally similar to the same region in $\Psi^{3WJ\text{-}1}$. Secondary structure of $\Psi^{3WJ\text{-}1}$ (*A*) and Ψ^{HP} (*B*). Non-native residues are shown in red. (*C*) 2D NOESY spectra overly of fully protonated Ψ^{HP} (green) and $A^{2r}G^{r}U^{r}$-labeled $\Psi^{3WJ\text{-}1}$ (blue). (*D*) 2D NOESY spectra overly of fully protonated Ψ^{HP} (green) and fully protonated $\Psi^{3WJ\text{-}1}$ (blue).

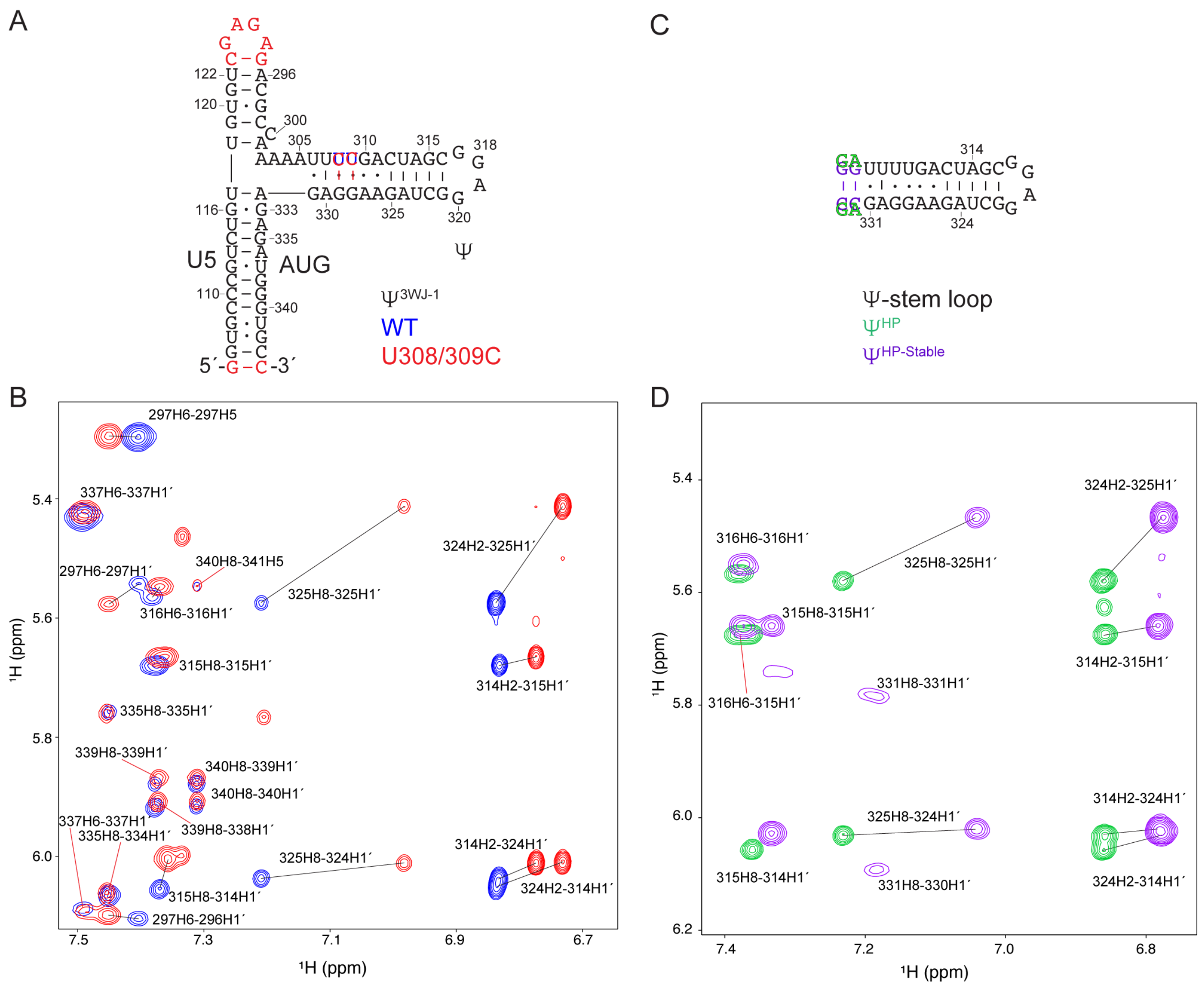

Fig. S6. Structural effects of stabilizing the [UUUU]:[GGAG] region. (*A*) Secondary structure of wild-type and G:U to G-C mutant of Ψ^{3WJ-1}. Non-native residues are shown in red. (*B*) 2D NOESY spectra overly of fully protonated wild-type (blue) and G:U to G-C mutant (red) Ψ^{3WJ-1}. (*C*) Secondary structure of Ψ^{HP} and $\Psi^{HP\text{-stable}}$. (*D*) 2D NOESY spectra overlay of fully protonated Ψ^{HP} (green) and $\Psi^{HP\text{-stable}}$ (purple).
